# Larval exposure to a pyrethroid insecticide and competition for food modulate the melanisation and antibacterial responses of adult *Anopheles gambiae*

**DOI:** 10.1038/s41598-020-58415-7

**Published:** 2020-01-28

**Authors:** Gaël Hauser, Jacob C. Koella

**Affiliations:** Institute of Biology, University of Neuchâtel, Rue Emile-Argand 11, 2000 Neuchâtel, Switzerland

**Keywords:** Malaria, Entomology, Immunology, Ecological epidemiology

## Abstract

The insecticides we use for agriculture and for vector control often arrive in water bodies, where mosquito larvae may be exposed to them. Not only will they then likely affect the development of the larvae, but their effects may carry over to the adults, potentially affecting their capacity at transmitting infectious diseases. Such an impact may be expected to be more severe when mosquitoes are undernourished. In this study, we investigated whether exposing larvae of the mosquito *Anopheles gambiae* to a sub-lethal dose of permethrin (a pyrethroid) and forcing them to compete for food would affect the immune response of the adults. We found that a low dose of permethrin increased the degree to which individually reared larvae melanised a negatively charged Sephadex bead and slowed the replication of injected *Escherichia coli*. However, if mosquitoes had been reared in groups of three (and thus had been forced to compete for food) permethrin had less impact on the efficacy of the immune responses. Our results show how larval stressors can affect the immune response of adults, and that the outcome of exposure to insecticides strongly depends on environmental conditions.

## Introduction

The immune system of mosquito vectors underlies their susceptibility to parasites, and thus their ability to transmit pathogens to humans^1,2^. While the strength of their immune response has a strong genetic component^3^, it is also influenced by the environment. Adult *Anopheles gambiae*, for example, have a less effective melanisation response if they were undernourished as larvae^4^. Correspondingly, the ability to transmit pathogens is influenced by the environment. Examples are that vectorial competence is influenced by the bacterial microbiota the mosquitoes acquire as larvae^5^, and that the susceptibility of *Aedes* mosquitoes to arboviruses and of *Anopheles* mosquitoes to malaria parasites depend on the temperature and food conditions during larval development^6–8^.

One aspect of the environment that is becoming increasingly important is the presence of insecticides. Because insecticides are used extensively in agriculture and vector control, they are often found in water of agricultural areas^9^, where mosquito larvae are exposed to them. Although their concentration is often so low that they do not kill the larvae, they affect the mosquitoes’ development. In particular, their effects can carry over to adults to influence their life- history traits (reproductive success, adult longevity, sex ratio^10–13^) and their vectorial competence for arboviruses^14,15^ and malaria^16,17^.

Such effects of sub-lethal doses of insecticides on vectorial competence are likely to be linked to their impact on the immune response. Indeed, exposure to insecticides affects the immune response of insects in several ways^18^: botanical insecticides^19,20^, an insect growth regulator^21^ and a pyrethroid^22^ decrease the activity of phenoloxidase (which is involved in the melanisation immune response) and botanical insecticides^23^, organophosphates and organochlorines^24^ affect the number of hemocytes (which determines the strength of phagocytosis).

It is, however, not known whether exposure of larvae will carry over to affect the immune response of adults. The goal of our project was to test whether such a carry-over exists. We therefore investigated the effect of exposing mosquito larvae to a field-realistic concentration of the pyrethroid permethrin, an insecticide widely used for agriculture and vector control, on the melanisation and antibacterial immune responses of adults. (i) The melanisation response of mosquitoes helps to clear many pathogens^25,26^ including malaria parasites^27,28^. A strong melanisation response against *Plasmodium* ookinetes may even lead to complete refractoriness against infection in *Anopheles gambiae*^29^. The melanisation response is a single component of the insect’s immune response. (ii) In contrast, the ability of the insect to suppress the growth of bacteria can be a consequence of several branches of the immune response and involves antibacterial compounds such as defensin, the use of reactive oxygen species, phagocytosis and the melanisation response^26^. These responses can also be effective against non-bacterial pathogens. Reactive oxygen species and phagocytosis, for example, are also involved in the mosquitoes’ defense against *Plasmodium*, for reactive oxygen species neutralize gametocytes and ookinetes^30,31^ and phagocytosis eliminates sporozoites^32^.

Since larval food affects many aspects of the mosquito’s life-history^33^, including vectorial competence^7,8^ and immune responses^4^, we expected that any effect of the insecticide would be stronger. We therefore compared its effects for well-fed larvae and for larvae that were forced to compete for food. Since larval competition and exposure to an insecticide may interact to affect the rate of larval development and the size of adults^34^, which in turn affect immunocompetence^4,35^, we also assessed the effects of our larval treatments on development time, larval survival and adult size.

## Results

### Larval development and adult size

Larval mortality ranged from 2.9% to 13.4% (Supplementary Figure S1). Permethrin increased mortality from 2.9% (95% CI: 1.9 to 4.5%) to 8.1% (6.3 to 10.3%) in individually reared larvae and from 3.9% (2.4 to 6.2%) to 13.4% (10.5 to 16.9%) in larvae reared in competition. While permethrin (χ^2^ = 49.07, df = 1, p < 0.001) and competition (χ^2^ = 9.36, df = 1, p = 0.002) significantly increased mortality, there was no significant interaction between the two factors (χ^2^ = 0.52, df = 1, p = 0.472). There was no significant difference among blocks (χ^2^ = 5.21, df = 1, p = 0.074).

Age at pupation ranged from 7 to 13 days. Larval competition increased the average age at pupation from 7.95 ± 0.04 (mean ± 95% CI) to 9.13 ± 0.06 days (χ^2^ = 340.42, df = 1, p < 0.001)(Supplementary Figure S2). Permethrin increased the age at pupation of individually reared larvae from 7.80 ± 0.04 days to 8.10 ± 0.05; contrast analysis: z = −9.57, p < 0.001), but did not affect the age at pupation of larvae reared in competition (9.11 ± 0.10 days vs. 9.15 ± 0.08, contrast analysis: z = 0.43, p = 0.67)(Supplementary Figure S2). Thus, the main effect of exposure to permethrin was not significant (χ^2^ = 0.18, df = 1, p = 0.669), but its interaction with competition was (χ^2^ = 37.25, df = 1, p < 0.001).

### Wing length

Whereas competition reduced wing length from 3.18 ± 0.02 mm (mean ± 95% CI) to 2.83 ± 0.02 mm (χ^2^ = 850.81, df = 1, p < 0.001), larval exposure to permethrin slightly increased wing length from 2.98 ± 0.03 to 3.01 ± 0.03 mm (χ^2^ = 5.62, df = 1, p = 0.018) (Supplementary Figure S3). There was no interaction between larval exposure and competition (χ^2^ = 0.07, df = 1, p = 0.784).

### Melanisation

We inoculated 582 mosquitoes, of which 42 died in the first 24 hours and were not included in the melanisation assay. The mortality after inoculation was about twice as high if the larvae had been reared in competition (9.3% (95% CI: 6.7 to 12.9%)) than if they had been reared individually (4.7% (95% CI: 2.8 to 7.9%)) (χ^2^ = 5.88, df = 1, p = 0.015). The mortality was affected neither by larval exposure to permethrin (χ^2^ = 0.90, df = 1, p = 0.343) nor by the interaction between the two factors (χ^2^ = 0.07, df = 1, p = 0.789).

**Figure 1:**
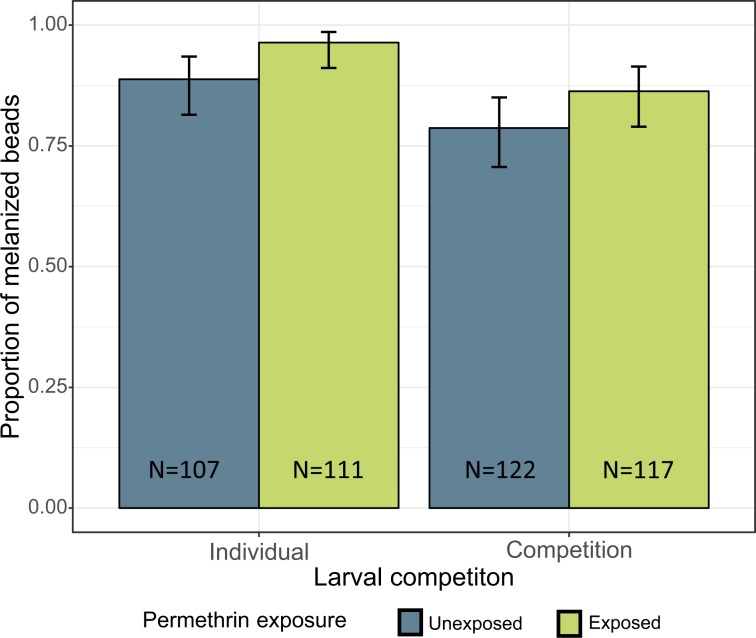
Melanisation success of the beads injected into adult *Anopheles gambiae* according to larval treatments. The mosquitoes were inoculated 4 days after emergence, and beads were recovered 24 h after the injection. The figure shows the proportion of beads that were at least partially covered by melanin according to larval competition and exposure to permethrin. Error bars show the 95% confidence intervals.

The injected bead was recovered in 457 of the remaining 540 mosquitoes; 399 of these were at least partially covered by melanin. Mosquitoes reared in competition were less likely to melanise their bead (82.4% (95% CI: 77.1 to 86.7%)) than those reared individually (92.7% (88.4 to 95.4%) (χ^2^ = 4.41, df = 1, p = 0.036, Fig. 1)). Mosquitoes exposed to permethrin as larvae were more likely to melanise their bead (91.2% (86.8 to 94.2%)) than unexposed ones (83.4% (78.0 to 87.7%)) (χ^2^ = 5.77, df = 1, p = 0.016, Fig. 1)). The interaction between exposure and competition was not significant (χ^2^ = 1.23, df = 1, p = 0.267).

**Figure 2:**
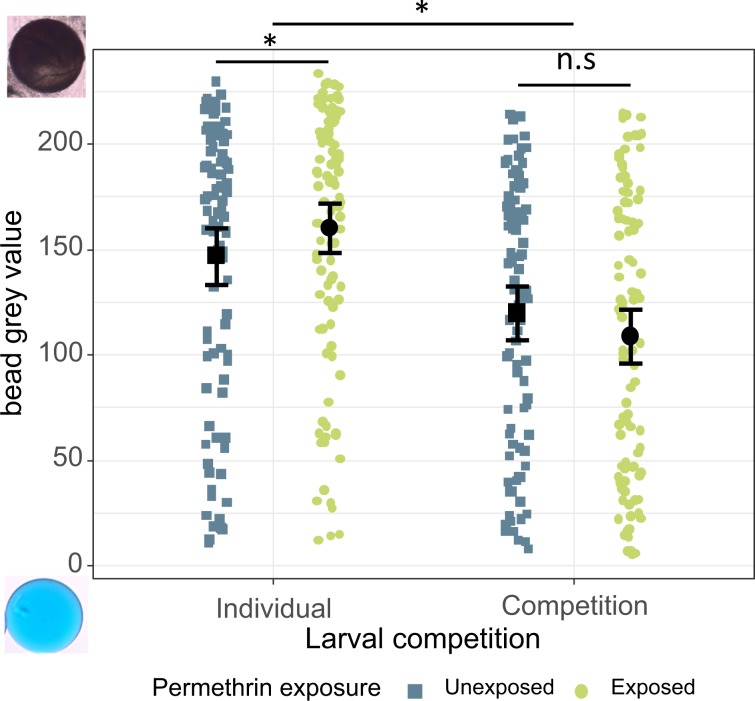
Degree of melanisation of the beads injected into adult *Anopheles gambiae* according to larval treatments. The mosquitoes were inculated 4 days after emergence, and beads were recovered 24 h after the injection. The figure shows the amount of melanin deposited on beads (unmelanised beads are excluded). A grey value of 0 would mean that the bead is unmelanised, while a grey value of 256 means that the bead is black and thus completely melanised. The two pictures on the left give examples of an unmelanised (bottom left) and melanized (top left) bead. The sample sizes are, from left to right: N = 95, N = 107, N = 96, and N = 101. Error bars show the 95% confidence intervals of the means.

In mosquitoes that had melanised their bead at least partially, the amount of melanin – estimated by the mean grey value of the bead – was affected by the larval treatments. Beads in mosquitoes reared in competition had less melanin (grey value of 114.1 ± 9.0 (mean ± 95% CI)) than those in individually reared mosquitoes (153.8 ± 8.9) (χ^2^ = 3.99, df = 1, p = 0.046). Larval exposure had no effect on the degree of melanisation (χ^2^ = 1.76, df = 1, p = 0.185), but there was an interaction between competition and larval exposure (χ^2^ = 5.54, df = 1, p = 0.019, Fig. 2). Contrast analysis showed that in individually reared mosquitoes permethrin increased the amount of melanin deposited on beads (from 146.9 ± 13.6 to 160.0 ± 11.9) (t = 2.00, df = 393, p = 0.046, Fig. 2), whereas in the competition treatment there was no effect of the insecticide (119.8 ± 12.7 for unexposed vs. 108.7 ± 12.9 for exposed mosquitoes) (t = −1.33, df = 393, p = 0.185), Fig. 2). The two experimental blocks led to similar patterns in our results for both the proportion of melanised beads and the degree of melanization.

### Antibacterial response

We inoculated 195 mosquitoes with *E. coli*, of which 8 died within 24 hours. The mortality was independent of larval competition (χ^2^ = 0.60, df = 1, p = 0.44) and of exposure to permethrin (χ^2^ = 0.00, df = 1, p = 0.98).

**Figure 3:**
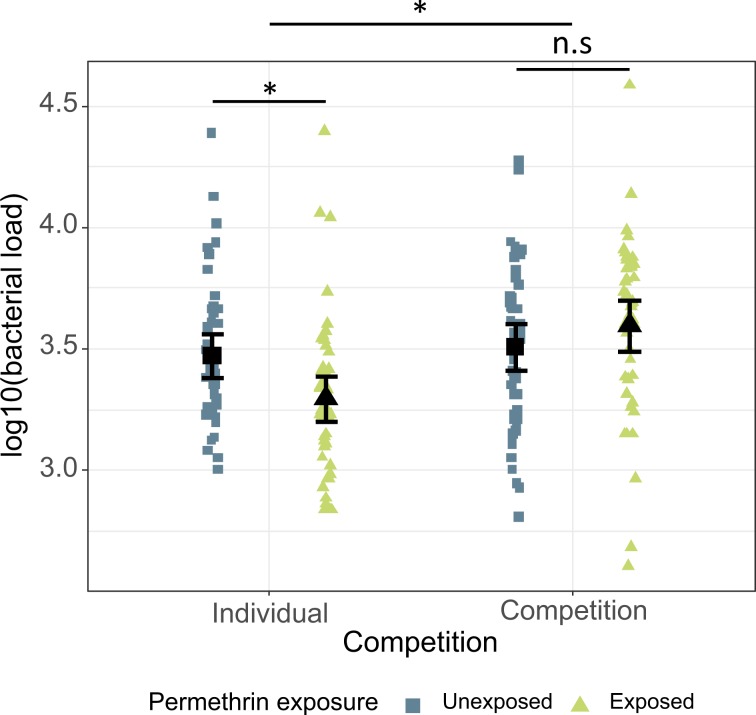
Bacterial load in adult *Anopheles gambiae* inoculated with *E. coli* bacteria according to larval treatments. The mosquitoes were inculated 4 days after emergence. The figure shows the number of bacteria (log10 transformed) retrieved from mosquitoes 24 h after inoculation. The sample sizes are, from left to right: N = 45, N = 47, N = 48, and N = 47. Error bars show the 95% confidence intervals of the means.

The bacterial load 24 hours after inoculation was higher in the competition treatment (4797.5 ± 963.7 (mean ± 95% CI)) than in the individually reared mosquitoes (3379.1 ± 831.6) (χ^2^ = 15.95, df = 1, p < 0.001), but there was no main effect of larval exposure to permethrin (χ^2^ = 1.70, df = 1, p = 0.193). However, there was a significant interaction between the two factors (χ^2^ = 7.83, df = 1, p = 0.005): in individually reared mosquitoes, larval exposure decreased the number of bacteria (from 3941.3 ± 1227.1 in unexposed to 2840.8 ± 1150.0 in exposed mosquitoes) (t = −2.667, df = 180, p = 0.008, Fig. 3), while it had no effect on mosquitoes reared in competition (4243.3 ± 1053.5 in unexposed vs. 5363.4 ± 1651.4 in exposed mosquitoes) (t = 1.284, df = 183, p = 0.201, Fig. 3).

## Discussion

Insecticide residues are frequently found in mosquito breeding sites, especially around agricultural areas. Using a field-realistic dose of permethrin that was sublethal for mosquitoes, we showed that the insecticide affects the life-history of the malaria vector *Anopheles gambiae* and, in particular, that it enhances the efficacy of its immune response once it emerges as an adult. We further showed that several effects of the insecticide are less apparent if mosquitoes competed for food during their development.

Mosquitoes that were reared in groups and thus forced to compete for food responded as expected^8,33^: they were more likely to die as juveniles, pupated later, and developed into smaller adults than individually reared mosquitoes. They also had a weaker immune response: they were less likely to melanise beads, deposited less melanin on the beads, and were less able to slow the replication *E. coli*. This corroborates the suggestion that the mosquito’s immune responses are energetically costly, so larval undernourishment limits the mosquitoes’ ability to defend themselves against pathogens^36,37^.

Mosquitoes that were exposed to a low dose of permethrin were also more likely to die and pupated later. However, they developed into slightly larger adults than unexposed larvae, as has been found for *Aedes aegypti* exposed to malathion^38^ or spinosad^39^. A possible explanation in the latter studies is that the death of some of the larvae reduced competition for food, so that the survivors obtained more food and grew larger. In our experiment, however, the effect was found for individually reared mosquitoes. As an alternative explanation, we therefore suggest that permethrin is more likely to kill the smallest, weakest larvae, while the larger ones are more likely to survive to become adults.

That larvae exposed to permethrin developed stronger immune responses as adults could be explained in at least two ways. First, as just discussed the exposure could let only the strongest individuals, so those with the most effective immune responses survive. Second, detoxifying insecticides may use processes that are also involved in the immune response. For example, exposure to insecticides leads to the long-lasting expression of genes, like defensin, that are involved in the mosquito’s immune responses^38^. Furthermore, several studies reported links between metabolic resistance to insecticides and immunocompetence: phenoloxidase (an enzyme that plays a central role in melanisation^27^ and contributes to antibacterial defense^40^) is more active in resistant *Culex pipiens* mosquitoes^41^ and *Plutella xylostella* moths^42^ than in sensitive ones, several antimicrobial peptides (AMP) are expressed more strongly in resistant *Culex pipiens* and *Anopheles gambiae*^43,44^, and nitric oxide synthase is expressed more in resistant than in sensitive *Anopheles stephensi*^45^.

That mechanisms are shared could, in turn, be explained in two ways. Genes that are involved in detoxifying insecticide can have a pleiotropic effect on immune responses. This may indeed be the case for phenoloxidase, which is not only necessary for the melanisation immune response, but may also play an active role in insecticide detoxification^42^. Alternatively, or additionally, a third physiological process could link detoxification and immune responses. One possibility is an interplay with reactive oxygen species, for permethrin induces a high level of oxidative stress^46^, which in turn increases melanotic encapsulation^47^ and antibacterial response^48^.

Despite the link between the detoxification of insecticides and the immune responses, and contrary to our expectation, most effects of permethrin were less apparent if larvae had been forced to compete than if they had been reared individually. As immunity and melanisation in particular are energetically costly^36,49^ and therefore involved in life-history trade-offs^50^, their activation likely depends on the resources available to mosquitoes. The observed results could thus be a direct consequence of limiting resources; when food is limited, pleiotropic effects may simply not be possible. It could also implicate the role of oxidative stress; with less food available, less energy is metabolized and less oxidative stress is generated^51,52^, which would weaken the above-mentioned link between detoxification and immune responses.

Together, our results show that insecticides in larval environment can have important carry-over effects on important mosquito defense mechanisms related to vectorial capacity. In particular, a stronger immune response decreases the intensity or the prevalence of an infection with *Plasmodium*, but also increase the mosquitoes’ longevity following the infection. Thus, while our study demonstrates the impact of an insecticide on mosquito’s immune response, further research is needed to precisely characterize the outcome of an infection with specific mosquito-borne pathogens. Finally, the obtained results also underline the role of larval competition for food in shaping the response of mosquitoes to insecticidal stress.

## Material and Methods

### Experimental design

We used the insecticide-sensitive Kisumu strain of *Anopheles gambiae s.s*^53^. to investigate the effects of a prolonged larval exposure to a sublethal dose of insecticide (permethrin) and of competition for food on two immune responses of adult mosquitoes: the melanisation response and the antibacterial response. The two immune responses were considered in separate experiments that used the same protocol to rear larvae and the same concentration of permethrin (as described below). In both, larvae were reared in a full factorial design, so that we could assess the combined effect of larval exposure and larval competition. We measured the melanisation response of adult mosquitoes by injecting Sephadex beads into their thorax and by measuring 24 hours after injection the amount of melanin that was deposited on the beads. For logistic reasons, we ran this experiment in two blocks with identical designs. We measured the response of adult mosquitoes against bacterial infection by injecting *E. coli* bacteria into their thorax and by assaying 24 hours later the survival of the mosquitoes and the number of bacteria.

The experiments were run in an insectary maintained at 26.5 ± 0.5 °C, 70 ± 5% humidity and a 12:12 light to dark photoperiod.

### Determination of sublethal dose of permethrin

In a preliminary experiment we determined a concentration of permethrin that (in our laboratory conditions) was sublethal for mosquito larvae yet ecologically relevant. An extensive review found the median concentration of permethrin residues in surface water is 0.04 µg/L (25^th^ percentile: 0.01 µg/L, 75^th^ percentile: 0.31 µg/L)^54^. We tested five concentrations of permethrin: 0.04, 0.1, 0.15, 0.3 and 0.8 µg/L. Permethrin solutions were made from a 1 µg/mL stock solution of solid permethrin (Sigma-Aldrich, Inc., St. Louis, Missouri) dissolved in pure ethanol. Between 40 and 50 larvae per concentration were reared individually in glass petri dishes (4 cm diameter x 1.2 cm height) containing 4 mL of 0.004% to 0.08% (volume per volume) ethanol containing the desired concentration of permethrin. (We had earlier found that a concentration of 0.08% ethanol induced no significant mortality). Among these concentrations, the highest one that caused no significant mortality was 0.1 µg/L (with a corresponding ethanol concentration of 0.01% v/v (78.9 µg/L). In our further experiments we therefore exposed mosquitoes to 0.01% ethanol with 0.1 µg/L permethrin or to 0.01% ethanol (as the unexposed control).

### Mosquito rearing and permethrin exposure

Freshly hatched *A. gambiae* larvae (0–3 h old) were put into glass petri dishes (4 cm diameter) containing 4 mL of 0.01% ethanol supplemented or not with 0.1 µg/L permethrin. They were reared either individually or in groups of three (that is in a non-competitive or a competitive environment). Tetramin Baby fish food was provided daily according to the age of the larvae: 0.04, 0.06, 0.08, 0.16, 0.32 and 0.6 mg per petri dish for larvae aged 0, 1, 2, 3, 4, and 5 or more days, respectively^55^. (Thus, competition implied less food per larva.) Pupae were transferred individually to 50 mL Falcon tubes. If more than one female emerged from a petri dish containing three larvae, we selected one randomly for further analysis to ensure independence of data. Males and unselected females were discarded. The selected females were transferred to 21 × 21 × 21 cm cages according to their treatment and age (one cage per day of emergence and treatment), where they had constant access to a 6% sucrose solution. As noted above, the two experiments (melanisation or antibacterial responses assays) used the same protocol to rear the larvae.

### Melanisation response (first experiment)

Melanisation ability was tested by inoculating 4-day-old adult females with negatively charged carboxymethyl Sephadex C-25 beads (Sigma-Aldrich, Inc., St. Louis, Missouri), according to an established procedure^56^. We chilled female mosquitoes in a Falcon tube placed on crushed ice for 5 to 10 minutes, and then injected one bead (50–130 µm diameter) into the thorax of the mosquito with a glass microcapillary. Injected females were transferred to cages and were frozen 24 hours after injection. Mosquitoes were dissected in 0.1% methyl green colored solution, and pictures of recovered beads were taken with a microscope with 20× magnification.

We assessed the melanisation response qualitatively by determining visually whether a bead was unmelanised or was melanised to some degree, and we assessed it quantitatively by estimating the amount of melanin deposited on a bead with the software ImageJ v1.51^57^. For each image, the color spectrum of unmelanised parts of the bead was identified and filtered from the bead, so that most of the color on the beads was due to melanin. We then measured the *mean gray value* of each (filtered) bead, giving 0 for entirely white beads, and 256 for entirely black beads. We also estimated the size of the bead by measuring with ImageJ its diameter.

### Anti-bacterial response (second experiment)

We assessed the efficacy of mosquito’s antibacterial response by measuring the growth of ampicillin-resistant *E. coli* (*dh5 alpha* strain) within the mosquito. We prepared the injection doses by measuring the absorbance at a wave-length of 600 nm of ampicillin-resistant *E. coli* that had been grown overnight in LA (Luria-Bertani broth containing 150 µg/mL ampicillin) at 37 °C, and comparing this absorbance with a standard curve that we had defined from *E. coli* solutions with known concentrations. Serial dilutions were made until the absorbance corresponding to 17.5 × 10^6^ *E. coli* per milliliter (3′500 bacteria per injection) was reached. The solution was kept on ice during the manipulation to avoid further bacterial growth. A fresh solution was prepared every day of injection.

Four days after emergence, we chilled mosquitoes on ice for 2–5 min and injected 3′500 *E*. coli (0.2 µL of bacteria solution) into the thorax with glass microcapillaries. We kept the inoculated mosquitoes for 24 hours in 21 × 21 × 21 cm plastic cages and then assayed the proportion that survived and measured the bacterial load in the surviving mosquitoes. For the latter, mosquitoes were briefly chilled on ice, transferred to 1.5 mL Eppendorf tubes and crushed using micro-pestles in 200 µL of LA. The homogenate was diluted 20-fold in LA, and 100 µL of this dilution were spread on LA agar plates. The agar plates were incubated at 37 °C overnight, and bacteria colonies were counted. The number of *E. coli* colonies was used as a measure of bacterial load in the mosquitoes.

### Statistical analyses

All analyses included the effects of larval competition (individually reared vs reared in groups of three), larval exposure to permethrin (exposed vs unexposed), their interaction and (where appropriate) experimental block.

### Larval development (both experiments)

Since the experiments assessing the melanisation response and the antibacterial response used the same protocol to rear the larvae, we analysed the two experiments as three experimental blocks (two blocks of the first experiment and one block of the second experiment).

We analysed larval mortality with a Generalized Linear Model (GLM) with quasibinomial errors, where the mortality in each petri dish was set as the response variable (mortality being 1 or 0 for individually reared larvae, and 1, 0.67, 0.33 or 0 for larvae reared in groups of three). We analysed age at pupation with a Cox’s proportional hazard models from the *survival* library in R^58^. In the competitive environment (where three larvae were reared together), we used the average age of pupation of the larvae in each petri dish as the response variable. Petri dishes in which one or more larva died were censored on the day the first larva died.

### Melanisation response (experiment 1)

Adult mosquitoes that had been inoculated with a bead were assayed for their wing length, their survival after inoculation, the presence or absence of melanin around the beads, and the amount of melanin deposited.

Wing length was analysed with a Linear Model (LM), where the distance from the axillary incision to the tip of the wing was set as the response variable^59^. Survival and the proportion of melanised beads were analysed with a GLM with binomial distribution of errors. The amount of melanin deposited, so the grey value of the beads, was analysed with an LM that assumes a normal distribution of errors. To reach normality of residuals, the square-root of the difference between the highest grey-value observed in our experiment (233.4) and the grey value of the bead was used. For both melanisation analyses, bead size was used as a covariate.

### Antibacterial response (experiment 2)

Survival after inoculation was analysed with a Generalized Linear Mixed-Effects model (GLMM) with binomial distribution of errors. The effect of competition or larval exposure to permethrin on the number of bacteria found in mosquitoes 24 h after the injection was tested with a Linear Mixed-Effects Model (LMM). The response variable was log-transformed to reach normality of residuals. Since the bacterial stock solution was made fresh every day, we included the day of injection as a random factor.

All analyses were performed with the software R (version 3.6)^60^. Statistical significances of the tested variables (larval competition and larval exposure to permethrin) were assessed with the Anova function of the *car* library^61^, using a type III anova if an interaction was significant, and a type II anova otherwise. For Linear (Mixed-Effects) Models the normality of residuals was checked visually, and homoscedasticity was tested with a Breusch-Pagan test (*bptest* function of the *lmtest* library^62^ in R). If interactions were significant, we performed contrast analyses between the factors of interest using *emmeans* (computing Estimated Marginal Means (EMM)) and *pairs* functions of the *emmeans* library in R, with p-values being adjusted using the *mvt* method.

## Supplementary information

Supplementary Information.

Supplementary Information.

## Data Availability

All data generated or analysed during this study are included as Supplementary Information files.
