# Supplementary material for: Larval exposure to a pyrethroid insecticide and competition for food modulate the melanisation and antibacterial responses of adult Anopheles gambiae
Source: Sci Rep. 2020 Jan 28;10:1364. doi: 10.1038/s41598-020-58415-7 (PMC6987095; doi:10.1038/s41598-020-58415-7)
Supplement: Supplementary file 2 — Supplementary Information. [file 41598_2020_58415_MOESM2_ESM.pdf]

Larval exposure to a pyrethroid insecticide and competition for food modulate the melanisation and antibacterial responses of adult *Anopheles gambiae*

***Authors:* Gaël Hauser, Jacob C Koella**

Supplementary Figures

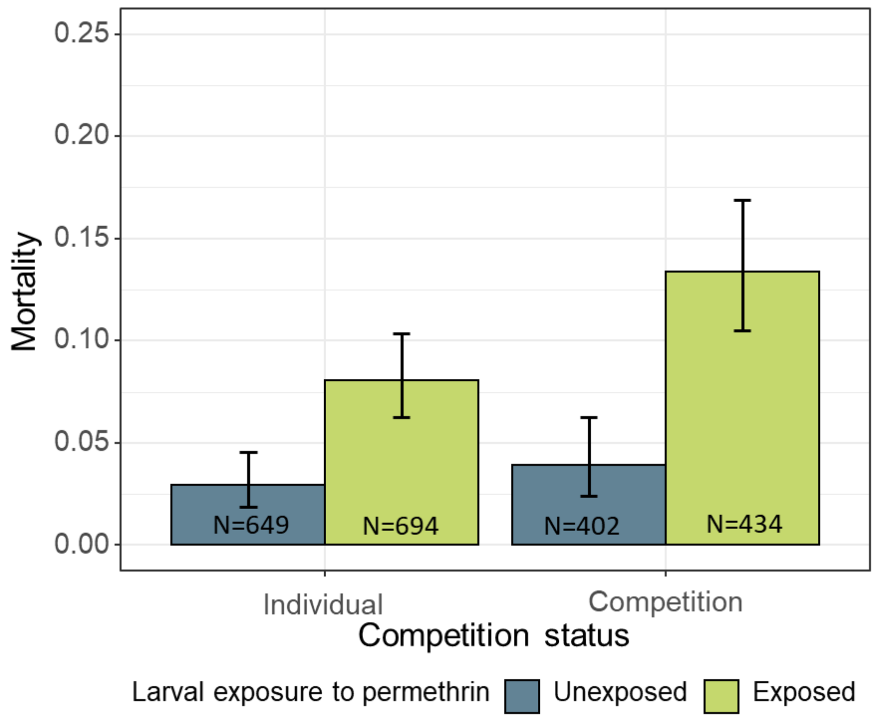

Figure S1. Mortality of the larvae during their development according to larval treatments: exposure to insecticide (unexposed vs exposed) and larval competition (individual vs competition (reared in groups of three)). The error bars represent the 95% confidence intervals.

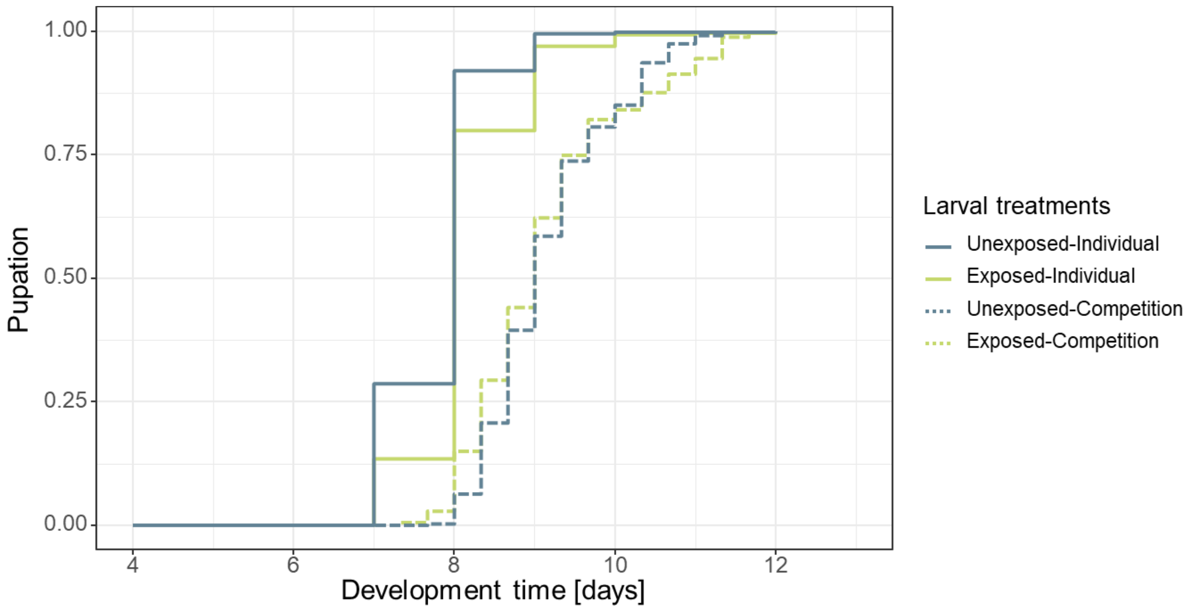

Figure S2. Development times of mosquito larvae according to the larval treatments: exposure to permethrin during development (exposed vs unexposed) and larval competition for food (individual vs competition (reared in groups of three larvae)). The sample sizes were (in order of the legend): N=649, N=694, N=434, and N=402. We analysed development time with a Cox Proportional Hazard model to be able to include larvae that died before pupation (which were censored in the model).

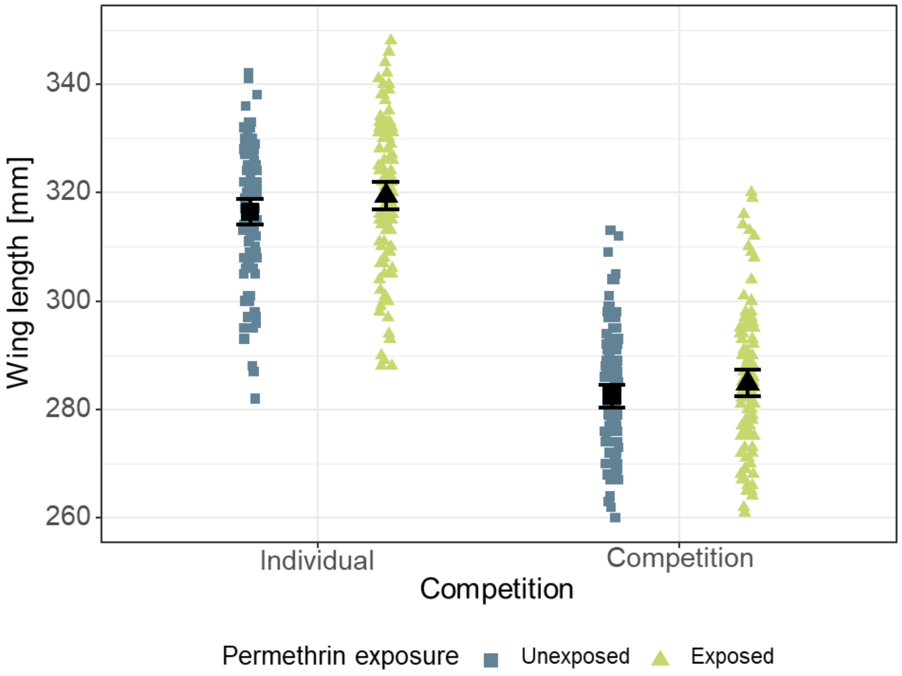

Figure S3. Wing lengths of mosquitoes according to larval treatments: exposure to permethrin during development (exposed vs unexposed) and larval competition for food (individual vs competition (reared in groups of three larvae)). Only mosquitoes of the melanization experiment are included, for the mosquitoes from the antibacterial response experiment had to be crushed and their wings could not be recovered. The sample sizes are (from left to right): N=106, N=111, N=117, and N=117. The error bars show the 95% confidence intervals of the means.
